# Deadly fraud – Food allergen substitution in the food chain

**DOI:** 10.1186/2045-7022-5-S3-P137

**Published:** 2015-03-30

**Authors:** Michael Walker, Hazel Gowland

**Affiliations:** 1 LGC/Safefood, Belfast, United Kingdom; 2 Allergy Action, St Albans, United Kingdom

## 

Food allergy, a significant public health concern in the developed world has a forensic context. Allergy related personal injury, fatality or criminal non-compliance with food law on the part of a food supplier are issues that have come before the UK courts. More recently substitution of peanuts for more expensive almonds has been discovered. Substitution, accidental or deliberate, subverts avoidance of allergens sometimes with deadly consequences. A common problem in fatal and near fatal reactions to such dishes is that customers are often a regular at a restaurant or takeaway, where their allergy is well known to staff. Sudden, unexpected reactions in respected and reputable businesses suggest fraud in the supply chain. The authors describe cases that arose from contraventions of food law, together with health and safety and civil litigation following deaths from food allergy. The cases we have looked at represent a spectrum, from unintentional or perhaps corner cutting to intentional, even criminal, addition of undisclosed allergens in food. But all jeopardise those with allergies and undermine the food industry's determination to manage allergens in a responsible manner. The Elliott Review - which many people may associate with the horsemeat scandal - actually looks into the integrity and assurance of food supply networks as a whole. Elliott recommends: "In sectors where margins are tight and the potential for fraud is high, even minor dishonesties must be discouraged and the response to major dishonesties deliberately punitive". We call for thorough investigation of food allergy deaths - particularly in the catering sector where there seems growing evidence that deadly fraud has infiltrated.

